# The proper strategy to compress and protect plasmid DNA in the Pluronic L64-electropulse system for enhanced intramuscular gene delivery

**DOI:** 10.1093/rb/rby028

**Published:** 2019-01-31

**Authors:** Yutong He, Yili Liu, Zhe Sun, Fei Han, James Zhenggui Tang, Rong Gao, Gang Wang

**Affiliations:** 1 National Engineering Research Center for Biomaterials; 2 Key Laboratory for Bio-Resource and Eco-Environment of Ministry Education, College of Life Science, Sichuan University, No. 29, Wangjiang Road, Chengdu, Sichuan, P.R. China; 3 Research Institute in Healthcare Science, Faculty of Science and Engineering, School of Pharmacy, University of Wolverhampton, Wolverhampton, UK

**Keywords:** muscle-based gene delivery, gene therapy, EGCG, PEI, Pluronic L64-electropulse

## Abstract

Intramuscular expression of functional proteins is a promising strategy for therapeutic purposes. Previously, we developed an intramuscular gene delivery method by combining Pluronic L64 and optimized electropulse, which is among the most efficient methods to date. However, plasmid DNAs (pDNAs) in this method were not compressed, making them unstable and inefficient *in vivo*. We considered that a proper compression of pDNAs by an appropriate material should facilitate gene expression in this L64-electropulse system. Here, we reported our finding of such a material, *Epigallocatechin gallate* (EGCG), a natural compound in green teas, which could compress and protect pDNAs and significantly increase intramuscular gene expression in the L64-electropulse system. Meanwhile, we found that polyethylenimine (PEI) could also slightly improve exogenous gene expression in the optimal procedure. By analysing the characteristic differences between EGCG and PEI, we concluded that negatively charged materials with strong affinity to nucleic acids and/or other properties suitable for gene delivery, such as EGCG, are better alternatives than cationic materials (like PEI) for muscle-based gene delivery. The results revealed that a critical principle for material/pDNA complex benefitting intramuscular gene delivery/expression is to keep the complex negatively charged. This proof-of-concept study displays the breakthrough in compressing pDNAs and provides a principle and strategy to develop more efficient intramuscular gene delivery systems for therapeutic applications.

## Introduction

It is an attractive strategy to produce therapeutic molecules using myocytes in skeletal muscles through intramuscular delivery of functional genes [1]. In this kind of gene therapy trials, naked plasmids have distinct advantages over viral vectors, such as biosafety, simplicity and cost efficiency [2]. Although in the pioneer study, ‘naked’ plasmid DNAs (pDNAs) were introduced into myocytes to successfully express reporter genes, direct administration of pDNAs showed unsatisfying results in therapeutic trials because of its low gene delivery efficiency [3]. Thereafter, efforts have been dominantly focusing on increasing delivery efficiency of pDNAs. Plenty of studies have revealed that it is an appealing way to transfer plasmids by using physical assistance, such as ultrasound, microbubbles and the most widely used electroporation [2, 4–9]. Besides, it is also worth noting that some chemical reagents such as poly(*N*-vinyl-pyrrolidone), poly(vinyl alcohol) and especially Pluronics are capable of promoting the efficiency of intramuscular gene delivery [10–15]. However, each approach has its own disadvantages and limitations. For example, the commonly used physical methods like electroporation and ultrasound still show potential safety risks to the body as well as histological damage or/and functional deterioration of local tissue [16, 17]. More and more researchers are developing combined strategies to enhance the gene expression level and reduce tissue toxicity.

In a previous study, we created an efficient and safe intramuscular gene delivery method by combining Pluronic L64 and low-voltage electropulse [18]. In this procedure, Pluronic L64 could interact with the cell membrane, disturb its integrity and increase its permeability, which consequently facilitated the migration of pDNAs driven by the low-voltage electropulse across the permeabilized membrane. Moreover, L64 has been proven to have the ability to accelerate the escape of pDNAs from endosomes and lysosomes [19]. The L64-electropulse method is among the most efficient intramuscular gene transfer procedures to date. However, pDNAs were still ‘naked’ in this procedure because they did not interact with L64 molecules.

Naked nucleic acids can be degraded by nucleases easily and rapidly in both the extracellular matrix (ECM) and intracellular environments [20]. Meanwhile, naked DNA would be unsuitable for efficient transmembrane migration in comparison with its compressed nanoscale counterparts [21]. Therefore, we expect that the transgene expression level of the L64-electropulse method could be significantly improved if the pDNAs were properly compressed and protected by an appropriate material. To compress pDNAs, the dominant approach is the application of non-viral cationic materials as vectors or carriers [22]. These cationic materials intact with anionic nucleic acids via electrostatic interactions, and the final material/pDNA complexes are usually cationic nanoparticles with optimized size and shape for effective *in vitro* gene transfection. However, cationic particles have been widely confirmed to be ineffective for intramuscular gene delivery and expression [23–25]. This may be due to the completely different extracellular environment of cultured cells compared with that of skeletal myocytes [26]. Negatively charged ECM components could bind positively charged material/pDNA complexes after local administration, which might disturb their mobility in tissue or interaction with target cells, resulting in a poor *in vivo* gene delivery/expression result [27–29]. Thus, to prevent non-specific interactions with ECM components, we think that the material/pDNA complex with negative charge might be an effective approach for the L64-electropulse-mediated intramuscular gene delivery system. It is hypothesized that the lower material/pDNA ratio, compared with that for optimized cell transfection, might result in negatively charged nanoparticle, thus facilitating *in situ* gene transfer to myocytes. However, it is still uncertain if the reduced material/pDNA ratio may bring about effective compression of pDNAs, and more importantly, offer efficient expression of the transgene.

Herein, we considered that the development of another type of gene vector with negative charge or even neutral could be an alternative strategy for *in situ* gene transfer into skeletal muscles. *Epigallocatechin gallate* (EGCG), a principal and the most widely studied component of green tea catechins, seems to be an ideal candidate because of its properties [30]. First, EGCG with phenolic hydroxyl groups has shown strong afﬁnity to DNA via cooperative hydrogen bonds and hydrophobic interactions rather than electrostatic interactions [31–33]. Second, EGCG can penetrate or be absorbed into the lipid bilayer of cell membranes [34–36], thus facilitating the translocation of EGCG/pDNA complexes across the membranes. Finally, EGCG can inhibit the activity of some nucleases by blocking their active sites [37–39], which may further strengthen the protective effect on DNA against enzymatic degradation.

In this proof-of-concept study, we investigated if the EGCG-compressed pDNAs can significantly improve intramuscular gene expression based on the L64-electropulse method. As the typical cationic vector, polyethylenimine (PEI) was investigated as a control to explore the influence of the zeta potentials of PEI/pDNA complexes with different N/P ratios on the transgene expression level and tissue toxicity in skeletal muscles. We expect to set up an efficient intramuscular gene delivery/expression system, and more importantly, to find out critical factors determining the proper compression and protection of pDNAs in such muscle-based transgene systems.

## Materials and methods

### Materials

Pluronic L64 and branched polyethylenimine (bPEI, Mw = 25 kDa) were purchased from Sigma-Aldrich (St. Louis, MO, USA). Tea polyphenol powder (EGCG wt% ≥80%) was obtained from Wuxi Taiyo Green Power Co. Ltd. (Jiangsu, China). DNase I and DNase I reaction buffer were obtained from Invitrogen (Carlsbad, CA, USA). HBG buffer (HEPES 20 mM, pH 7.4, 5% (w/v) glucose) and other buffers were prepared in MilliQ ultrapure water and ﬁltered (0.22 μm) prior to use. All other chemicals were purchased from Sigma-Aldrich and used without further puriﬁcation. All chemicals and reagents were of analytical grade.

The plasmids encoding β-d-galactosidase (pCMV-LacZ), luciferase (pCMV-Luc), far-red ﬂuorescent protein (pCMV-E2) and growth-hormone-releasing hormone (pCMV-GHRH) were previously constructed in our laboratory [18, 23, 25]. The plasmids were ampliﬁed in *E. coli* DH5α and extracted using the Endo-Free Plasmid Kit (Invitrogen, Carlsbad, CA, USA).

### Preparation and characterization of PEI/pDNA polyplexes and EGCG/pDNA mixtures

The N/P ratio of PEI to pDNA is deﬁned as the molar ratio of nitrogen in PEI to phosphates in pDNA. PEI/pDNA polyplexes or EGCG/pDNA mixtures were prepared in HBG buffer by adding variable amounts of PEI or EGCG solutions into equal volume of solutions containing fixed amount of pDNAs. Each resultant formulation was incubated for 30 min at room temperature before use.

The nanoparticle size (diameter, nm), polydispersity index (PDI) and zeta potential (ζ) were measured on a Zetasizer Nano ZS (Malvern Instruments, Worcestershire, UK) dynamic light scattering (DLS) instrument. The samples were diluted to 1 ml with MilliQ water to a final DNA concentration of 3 µg ml^−1^. All measurements were performed in triplicate at 25°C. The morphology and size of particles were examined by atomic force microscope (AFM, MFP-3D-BIO, USA). Brieﬂy, samples were prepared at a ﬁnal DNA concentration of 20 µg ml^−1^ and then spread out on freshly cleaved mica sheets. After an overnight adsorption, samples were detected using AFM.

### DNase I protection assay

PEI/pDNA complexes or EGCG/pDNA mixtures containing 400 ng pDNAs (pCMV-Luc) were incubated with 0.5 U DNase I (1 U μl^−1^, Fermentas) at 37°C for 10 min in 10 µl solution. Samples were then treated with 5 µl of 25 mM EDTA and subsequently bathed at 65°C for 10 min to inactivate the enzyme. Six microlitres of heparin or urea (10 mg ml^−1^) were added to PEI/pDNAs or EGCG/pDNAs, respectively, and incubated for another 2 h at 37°C to release the pDNAs from complexes. Samples were then assessed by electrophoresis on a 1% (w/v) agarose gel for 45 min at 80 V. To investigate the inhibition of nuclease activity of EGCG, DNase I was pre-treated with EGCG for 10 min, and then pDNAs were added and incubated for 10 or 30 min, followed by the above steps including inactivation of the nuclease, release of the pDNA and running the gel electrophoresis to compare the intensity of DNA bands.

### Intramuscular transgene administration

All experiments with live animals were performed in compliance with the relevant laws and institutional guidelines of Sichuan University, which were approved by the ethics committee of Sichuan University. Four- or six-week-old male BALB/c mice were used to evaluate *in vivo* expression of GHRH or reporter genes, respectively. Ten micrograms of reporter plasmids (pCMV-LacZ, pCMV-Luc and pCMV-E2) or 25 µg of pCMV-GHRH in 40 µl solution were used for each intramuscular injection. PEI/pDNAs or EGCG/pDNAs were mixed with 0.4% (w/v) of Pluronic L64 diluted in saline to get the final concentration of 0.1% L64, kept for 5 min at room temperature and injected into each side of the tibialis anterior (TA) muscle using a 29-gauge BD ultra-fine insulin syringe (BD, Franklin Lakes, NJ, USA) for 2–5 s. The transcutaneous electrotransfer was subsequently administrated using previous protocol [18]. The different systems were constructed and named as L/E, L/E/P and L/E/G respectively. Herein, L refers to 0.1% (w/v) Pluronic L64, E = electrotransfer, P =PEI and G =EGCG.

### *In vivo* assays of reporter and functional genes expression

Experimental mice were sacriﬁced and muscle samples were isolated at the indicated days after injection. For the *in situ* β-galactosidase activity assay, tissues were fixed in the commercial ﬁxative for at least 20 min on ice. After three times of rinsing with PBS, fixed samples were stained using β-Galactosidase Staining Kit (Beyotime, Shanghai, China) for 6 h and then photographed with a digital camera. Luciferase activity was analysed using the Luciferase Reporter Assay Kit (Promega, Madison, WI) according to the manufacturer’s protocol. Briefly, each sample was homogenized in 1 ml ice-cold reporter lysis buffer and then frozen at −80°C overnight. The frozen tissues were thawed and centrifuged at 12 000 g for 3 min at 4°C. Twenty microlitre supernatant and 100 µl luciferase substrate were mixed in a 96-well plate and the luciferase activity was detected by measuring the light emission for 10 s in a spectral scanning multimode reader (Varioskan Flash; Thermo Fisher). BCA Protein Assay Kit (Pierce, Rockford, IL, USA) was used for measuring the protein concentration. The transgene efficiency was presented as relative light units per milligram of total protein (RLU/mg protein). To evaluate the expression of GHRH, the body weight of the mice was recorded and the concentration of GHRH in serum was measured using the GHRH ELISA kit (Yuduo Co. Ltd., China).

### The far-red ﬂuorescent protein expression assay

Expression of E2-Crimson far-red ﬂuorescent protein *in situ* was detected by *In-Vivo* Imaging System (CRI Maestro, Boston, USA) at days 7, 14 and 21 post-intramuscular injection. The legs of mice were thoroughly depilated and mice were anaesthetized with 5% (w/v) chloral hydrate liquid before imaging. The scanning wavelengths were ranged from 600 to 700 nm.

### Histological section preparation and analysis

TA muscles were separated and ﬁxed in 4% fresh neutral paraformaldehyde solution for 48 h, and then orderly dehydrated with gradient concentrations of ethanol and pure xylene. Well-dehydrated samples were embedded in parafﬁn, sliced in both cross-sections and longitudinal sections with 10 µm thickness by a rotary slicer. The sections of specimens were photographed by a microscope (Leica DMI 4000B; Wetzlar, Germany) after staining with haematoxylin and eosin.

### Statistical analysis

All data were reported as mean ± standard deviation (SD) of six independent samples. Statistical data analysis was performed by Student’s *t*-tests assuming a two-tailed distribution and the variance (ANOVA) was analysed to evaluate multiple comparisons using graphpad prism 5.0. Differences were considered statistically signiﬁcant at *P* values < 0.05.

## Results

### Zeta potential, size and morphology of PEI/pDNA and EGCG/pDNA complexes

**Figure 1.**
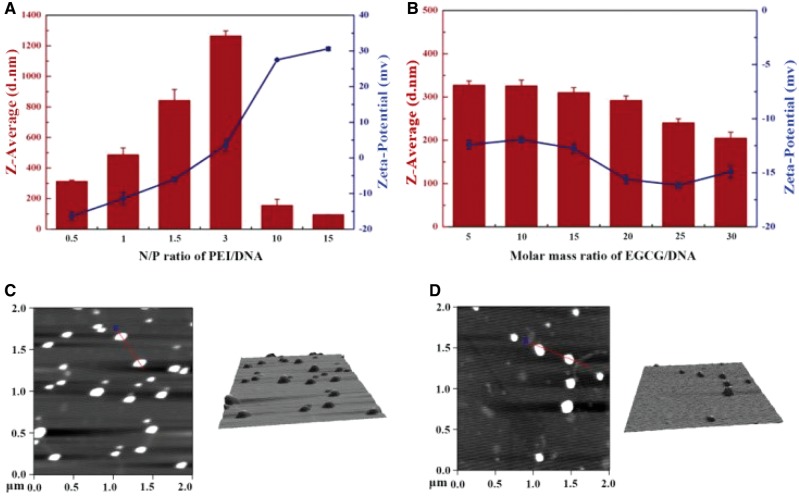
Characterization of PEI/pDNA polyplexes and EGCG/pDNA compounds. Zeta potential and size measurements via DLS of (A) PEI/pDNA complexes under various N/P ratios and (B) EGCG/pDNA compounds with given molar ratios. Morphology and size analysis by AFM of (C) PEI/pDNA at N/P = 0.5 and (D) EGCG/pDNA at molar ratio = 10

For PEI/pDNA polyplexes, the diameter and surface charge of the particles were N/P ratio dependent (Fig. 1A). The average size of the complex increased from 300 to 1200 nm at N/P ≤ 3, and decreased to smaller than 300 nm when N/P was ≥10. The zeta-potential value changed from about −15 mV to +30 mV with increasing N/P ratios. Therefore, the negatively charged nanoparticles with relatively small sizes were obtained, when N/P = 0.5 and 1. When N/P > 3, the average size sharply decreased and the zeta-potential turned to be positive.

For EGCG/pDNA complexes, the average size decreased smoothly and moderately from 330 to 200 nm while molar mass ratios increased from 5 to 30 (Fig. 1B). The zeta-potential of the EGCG/pDNA particles was negative, and varied slightly between −12.5 and −16 mV. Morphology analysis by AFM showed that both PEI/pDNA (N/P = 0.5) and EGCG/pDNA (molar ratio = 10) were well compressed to nanoscale with nearly spherical 3D architectures (Fig. 1C and D).

### Comparison of the protective effect of PEI and EGCG on pDNAs against DNase I degradation

**Figure 2.**
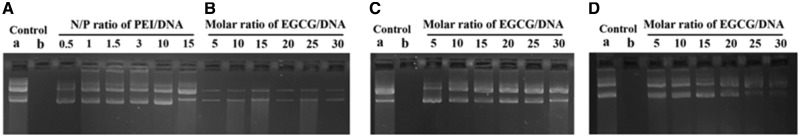
Protection of pDNAs from DNase I degradation by PEI or EGCG. Control a: naked pDNA. Control b: naked pDNA treated with DNase I. A and B: PEI/pDNA and EGCG/pDNA treated with DNase I for 10 min. DNase I pre-treated with EGCG for 10 min, and then incubated with pDNAs for (C) 10 min or (D) 30 min

An indispensable prerequisite for gene carriers is to provide protection to DNA against degradation. With a routine procedure, while DNase I was added after formation of carrier/pDNA complexes, pDNA bands in PEI/pDNA (Fig. 2A) and EGCG/pDNA groups (Fig. 2B) were detectable. It indicates that pDNAs were protected in both complexes. The differences in intensity of DNA bands between PEI groups and EGCG groups suggest that the two materials have different DNA protection efficiencies. PEI was more capable of protecting pDNAs from enzymatic degradation, which might be caused by the stronger compression effects of cationic materials on pDNAs. Figure 2C and D was used to explain the ability of enzymatic inhibition of EGCG. In an optimized procedure, EGCG was first mixed and pre-incubated with DNase I for 10 min and then added into pDNA solutions. The intensity of pDNA bands in EGCG groups was almost the same as that of untreated pDNA bands, indicating the ability of EGCG on DNase I resistance (Fig. 2C). The intensity of DNA bands weakened but still were seen clearly within the extended incubation time of 30 min (Fig. 2D), which implied the DNase I resistance effect of EGCG was not instantaneous. In comparison, PEI did not show such DNase I resistance unless it formed PEI/pDNA complexes (data not shown). The results revealed that EGCG and PEI had different ways to protect pDNAs. EGCG protected pDNAs by directly inhibiting the nuclease activity of DNase I as well as by binding DNA to form a stable and compact structure, whereas PEI depended on its compression capability of DNA.

### Efficiency and biocompatibility of L/E/P system in local gene delivery

**Figure 3.**
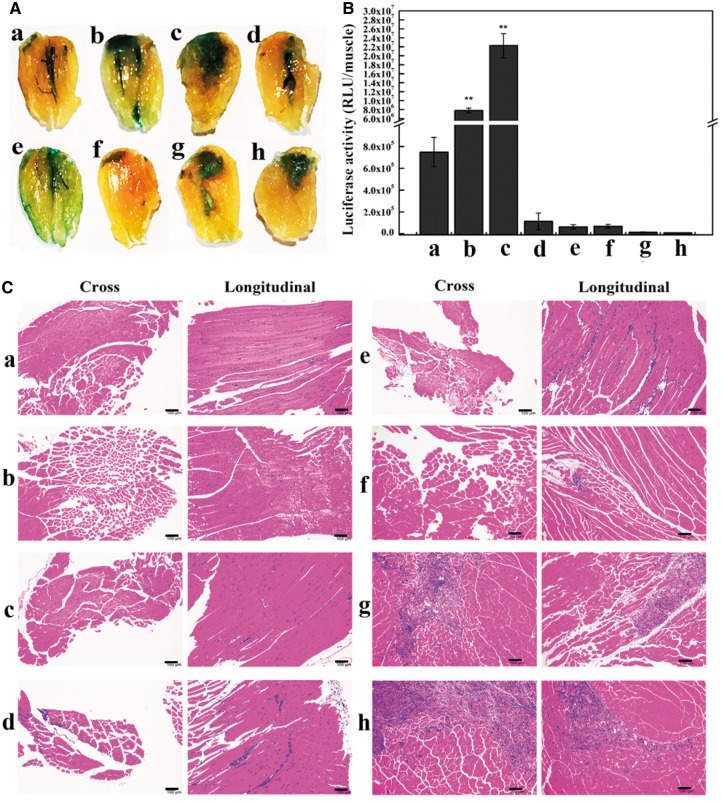
*In vivo* evaluation of transgene expression in L/E/P system and the histological analysis of the injected TA muscles. *n* = 6. Time: 7 days after injection. (A) Qualitative observation of β-galactosidase expression. (B) Quantitative detection of luciferase activity. (C) Representative H/E-stained tissue sections of the injected TA muscles. (a) Naked pDNA group; (b) L/E group; (c–h) L/E/P groups with various N/P ratios ranging from 0.5, 1, 1.5, 3, 10 and 15, respectively. **P < 0.01 vs. pDNA. Scale bar = 100 µm

In the L/E/P *in situ* gene delivery system, when pDNAs were condensed by PEI, the N/P ratio of PEI nitrogens to DNA phosphates was pivotal for the expression of reporter genes and the occurrence of cytotoxicity. The levels of β-galactosidase (Fig. 3A) and luciferase expression (Fig. 3B) at N/P ratio 0.5 (L/E/P-0.5) were significantly higher than that of the L/E group. However, both genes expression sharply decreased with N/P ratios of >0.5. Quantitatively, luciferase expression in L/E/P-0.5 increased to 29.7-fold or 2.9-fold as compared with that in the naked pDNA or L/E group, respectively. When the N/P ratio was increased to 1, 1.5 and 3, the gene expression was at least three orders of magnitude lower compared with those in the L/E/P-0.5 or even the L/E group. At N/P = 10 and 15, the gene expression has already reduced hundreds fold as compared with that in the naked pDNA group. The results demonstrate that the L/E/P formulation can enhance intramuscular gene transfer efficiency at a specific N/P ratio.

The potential damage on muscle tissues from different treatments was evaluated to account for *in vivo* biocompatibility. Cross- and longitudinal-section from the injected TA muscles were prepared for the histopathological analysis 7 days after treatment. As shown in Fig. 3C, no pathological changes were found in the L/E/P-0.5, naked pDNA and L/E groups. It indicated that binding nucleic acids with a small amount of PEI is safe for local transgene administration in skeletal muscles. As the N/P ratio increased, histological sections of muscle showed more infiltration inflammatory cells and myocyte damage. A large area of inflammatory cell infiltration and necrotic muscle ﬁbres appeared once N/P ratios were >10. Taken together, N/P ratio at 0.5 is the safe and most suitable condition for L/E/P approach in intramuscular application.

### *In vivo* evaluation and optimization of L/E/G system

**Figure 4.**
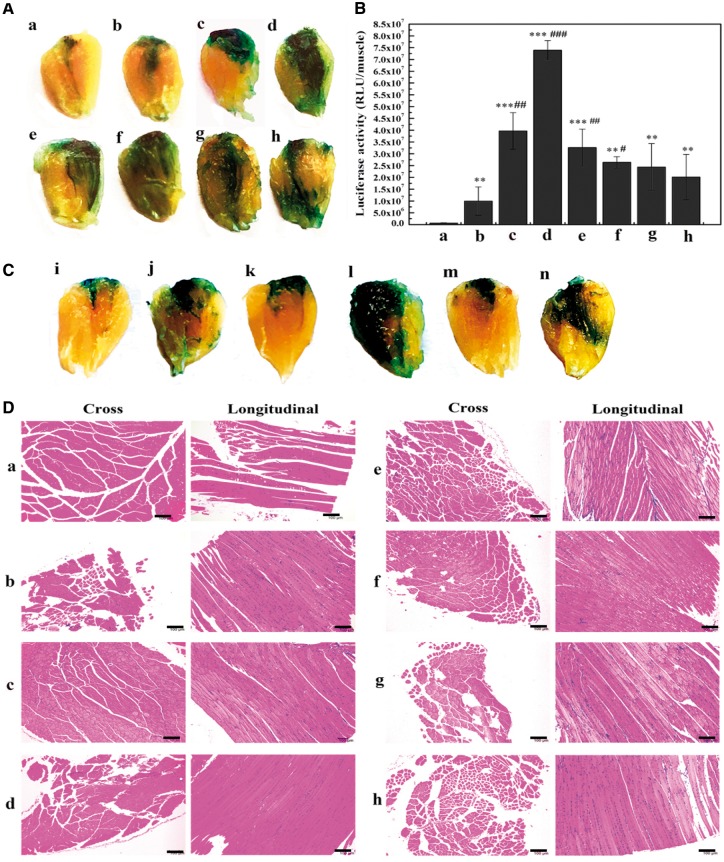
Evaluation of intramuscular transgene expression and histological analysis in L/E/G system. *n* = 6. Time: 7 days after injection. Representative images of (A) β-galactosidase expression or (B) luciferase activity assay in L/E/G treatment. (a) Naked pDNA group; (b) L/E group; (c–h) L/E/G groups with different molar ratios of EGCG/pDNA at 5, 10, 15, 20, 25 and 30. **P < 0.01 vs. pDNA, ***P < 0.001 vs. pDNA, ^#^*P* < 0.05 vs. L/E, ^##^*P* < 0.01 vs. L/E, ^###^*P* < 0.001 vs. L/E. (C) Qualitative evaluation of the necessity of G or L/E in L/E/G treatment. (i) Naked pDNA group; (j) L/E group; (k) G alone (molar ratio of EGCG/pDNA = 10); (l) L/E/G (molar ratio = 10); (m) G alone (molar ratio at 60); (n) L/E/G (molar ratio at 60). (D) Representative H/E-stained tissue sections of the injected TA muscles after different treatments as indicated in A and B. Scale bar = 100 µm

Negative charge characteristic and the traits of EGCG on bonding DNA and inhibiting enzyme activity inspired us to replace PEI with EGCG to construct an original EGCG-integrated L64-electropulse intramuscular gene transfer system (L/E/G). As shown in Fig. 4A, L/E/G groups with EGCG/pDNA in molar ratio from 5 to 30 provided stronger LacZ expression than those in naked pDNA and the L/E groups. This is evident from the LacZ staining and the distribution areas on the surface of TA muscles (Fig. 4A). Besides, LacZ expression level varied in different groups with the highest level occurring at the molar ratio 10 (L/E/G-10). Further quantitative analysis by luciferase activity assay confirmed that RLU values in L/E/G groups were obviously higher than that in the pDNA and L/E groups, and the maximum also occurred in the L/E/G-10 group, with a gradual decline thereafter (Fig. 4B). The highest luciferase expression quantitatively increased to 7.6 times as compared with that in the L/E group, and even 122 times to pDNA administration alone.

In addition, in the groups of EGCG/pDNA (without L/E) at molar ratio of 10 (Fig. 4C, k) or even as high as 60 (Fig. 4C, m), the β-galactosidase expression was slightly higher than that in naked pDNA group and was comparable to that in the L/E treatment (Fig. 4C, i or j). More importantly, β-galactosidase expression level was significantly improved when EGCG was introduced into the L/E method (Fig. 4C, l and n). It reveals that the combination of EGCG with L/E method generated synergistic effects on transgene delivery and expression, and EGCG should have significant role in the integrated L/E/G method for improving gene expression.

It is essential to consider biocompatibility of L/E/G system for *in vivo* application. Haematoxylin/eosin (H/E) staining was utilized to visualize the general or pathological morphologies of TA muscles treated with different formulas. The results of EGCG-containing groups (Fig. 4D, c–h), which had no pathological changes, revealed no visible differences with control groups (Fig. 4D, a and b).

**Figure 5.**
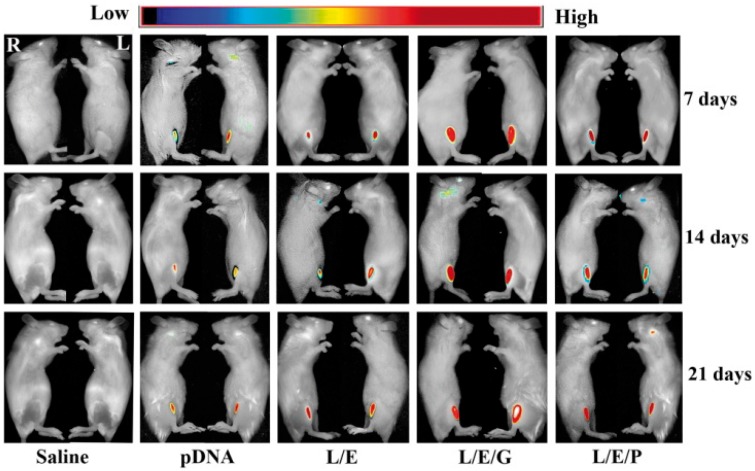
Comparison of intramuscularly expressed ﬂuorescent protein in different optimized formulas. L/E/G: molar ratio of EGCG/pDNA = 10 (L/E/G-10); L/E/P: N/P ratio of PEI/pDNA = 0.5 (L/E/P-0.5). *n* = 6

To investigate the intensity and duration of exogenous gene expression in living animals, the expression of the far-red ﬂuorescent protein E2-Crimson was observed in living mice at days 7, 14 and 21 after a single administration. In each group, fluorescent signals could be analysed for at least 3 weeks without any attenuation (Fig. 5). In terms of expression level, the L/E/G-10 exhibited the strongest signals among the four treatments while L/E/P-0.5 was slightly superior to naked pDNA and L/E groups. These results were consistent with those of luciferase and β-galactosidase expression. Therefore, all data reveal that the L/E/G method is more effective than L/E/P method in skeletal muscle-targeted gene delivery.

### Expression of functional gene using the optimized L/E/G method

**Figure 6.**
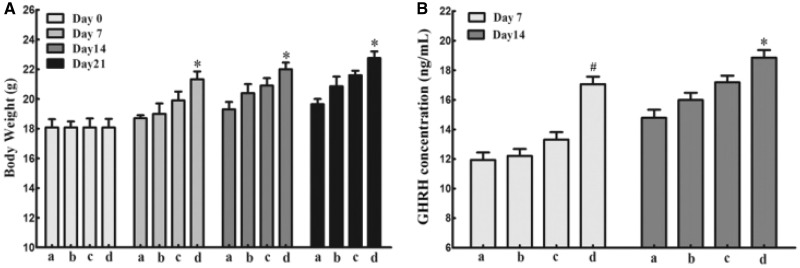
Intramuscular expression of the GHRH. (A) Average body weights. (B) Concentration of the GHRH in circulation. (a) saline group; (b) pDNA group; (c) L/E group; (d) L/E/G-10. *n* = 6. *P < 0.05 vs. groups a and b, ^#^*P* < 0.05 vs. other groups

Using skeletal muscle as a “factory” to produce therapeutic proteins is an attractive strategy for many diseases therapy. The expression of therapeutic genes should be high enough in targeted tissues or the circulatory system so that the biological or therapeutic results can be detectable or visible. Therefore, we investigated the applicable potentiality of L/E/G method via examining the expression of functional genes. Mouse GHRH can generate strong physiological effects on mouse growth, which can be easily detected by measuring the body weight of a mouse. After a single administration of 50 µg plasmid pCMV-GHRH per mouse, the body weights were recorded at days 7, 14 and 21. Comparing to the saline group, the other three groups showed faster growth rates to varying degrees at certain time. Among them, the optimized L/E/G-10 group exhibited the highest accelerated growth rate in the first 21 days after an intramuscular injection (Fig. 6A). At the same time, the serum concentration of serous GHRH in the L/E/G-10 treatment was higher than the other groups at day 7 and 14 (Fig. 6B). The results of GHRH expression demonstrate that the L/E/G-10 method has applicable potential on gene therapy.

## Discussion

The main objective for intramuscular gene expression is to ‘produce’ designed functional molecules, such as antibodies and enzymes, for specific purposes. For plasmid-based gene expression systems, the expression level or the amount of expressed target molecules is the most crucial consideration. The L64-electropulse method is one of the most efficient intramuscular gene delivery/expression methods to date, but the plasmids here are still ‘naked’. Unconsolidated and chemically unmodified structure features of naked DNA confer an inherently inadequate or ineffective cellular uptake profile and extremely low biological stability to *in vivo* applications.

Packaging pDNAs with gene carriers in the L/E system is identified as capable of overcoming these drawbacks, and consequently improving intramuscular gene expression. Cationic vectors are one of the most widely used materials to package anionic DNA for protecting nucleic acids against nuclease degradation and thus promoting gene transfer into cultured cells. However, the use of cationic vectors often has adverse side effects in intramuscular gene transfer trials. The poor results may be due to the different extracellular environments between *in vitro* cultured cells and *in vivo* myoblasts. For cultured cells, the relatively simple liquid extracellular environment allows cationic vector/pDNA particles to easily migrate and combine with cell membranes. *In vivo*, cationic vector/pDNA particles can be bound and trapped by the negative charge of ECM components after local administration into skeletal muscles, resulting in limited mobility to target cells and gene transfection. Negatively charged vector/pDNA particles may be a solution for this dilemma.

To address this, first PEI was selected to compress pDNAs. A series of PEI/pDNA particles were prepared with different sizes and surface charges (Fig. 1A). With the increase of N/P ratios from 0.5 to 3, PEI/pDNA particles enlarged rapidly and reached the peak at N/P = 3 (∼1300 nm, PDI = 1.0), suggesting that precipitation happened. At the same time, the value of zeta potential gradually decreased and approached neutral at N/P = 3 (Fig. 1A). The enlarged size restricted the translocation of the particles in the ECM and across cell membranes, while the neutralized charge weakened the interaction power between the particles and the electric field, which was considered to be the main force to drive the particles migrating across the permeabilized cell membranes. When the N/P ratio increased to 10 or 15, the PEI/pDNA particles were positively charged, making them to be easily trapped in the ECM and detrimental to the muscular tissue. In fact, the highest expression efficiency (Fig. 3A and B) and minimal tissue toxicity (Fig. 3C) were achieved at the same time at N/P ratio of 0.5 (L/E/P-0.5). Altogether, N/P ratio = 0.5 for PEI/pDNA is an optimal parameter for muscle-based L/E/P gene delivery system. The nanoscale, negative charge and regular morphology may cooperatively contribute to the positive performance of PEI/pDNA complexes at N/P = 0.5.

It has been proven that the N/P ratio among 10 and 15 is optimum for cell transfection when 25 kDa branched PEI is used. The complete binding of PEI with DNA usually fulfils at N/P ratio of 3 [40]. The redundant free PEI molecules can assist PEI/pDNA particles escape from endosomes via the mechanism of proton sponge effect. At N/P ratio of 0.5, the number of PEI molecules is insufficient to completely compress pDNAs and to timely release PEI/pDNA complexes from endosomes via the proton sponge effect. However, increasing the amount of PEI may bring out adverse side effects. Therefore, it seems that it is a paradox to use PEI in the L/E-based method. It is speculated that other cationic materials are up against the same situation.

In view of the dilemma of cationic materials, it is necessary to find another kind of material for the L/E procedure. An ideal alternative material should have the ability to effectively compress nuclear acids to form particles with negative charge, probably through an interaction manner other than electrostatic interactions. Here, we found that EGCG was such an ideal alternative. First, EGCG is biodegradable, and has a negative charge as well as a high affinity to DNA through cooperative hydrogen bond and hydrophobic interactions. pDNAs were efficiently compressed by EGCG, generating EGCG/pDNA particles ranging from 200 to 330 nm, which had suitable size distribution and nanoscale architecture for gene transfer into cells (Fig. 1B and D). Meanwhile, zeta potentials of all EGCG/pDNA particles were within −12 to −16 mV, avoiding them to being trapped in the negatively charged ECM environment in skeletal muscles via electrostatic interactions (Fig. 1B). Second, EGCG has the property of inhibiting nuclease activity, providing an extra protective effect on pDNAs against DNase I digestion. EGCG can protect pDNAs by compressing them and inhibiting DNase I activity simultaneously (Fig. 2B–D). Third, the structural features of EGCG allow high afﬁnity binding to lipid bilayers of biomembranes, which might augment cellular uptake of particles. Fourth, the anti-inflammatory effect of EGCG is speculated to reduce the local acute inflammation after injection of heterogenous substances, facilitating *in situ* expression of exogenous genes. Finally, as a food component, EGCG is safe enough for *in vivo* application. No pathological changes including inﬂammatory cell infiltration and muscle necrosis were found in all the EGCG-containing treatments (Fig. 4D). These ﬁndings suggest that the EGCG-integrated L/E/G method has great potential with credible biosafety for the application of intramuscular gene delivery.

The combination of all advantages of EGCG makes it a unique and potent integrant in the constructed L/E/G intramuscular gene delivery/expression method. The *in vivo* transgene expression by the method was assessed using both reporter and functional genes. The results of reporter genes expression qualitatively or quantitatively demonstrated that the highest expression level as well as the good biocompatibility (Fig. 4D) and duration (Fig. 5) were presented by the L/E/G-10 administration (Fig. 4A–C). At the same time, both the effects of GHRH on mouse growth and the serous GHRH concentration were significantly improved by the L/E/G-10 administration in the functional gene expression assay (Fig. 6). The results displayed the promising potential of the L/E/G-10 method for therapeutic purpose.

## Conclusion

Compressing pDNAs to proper architecture with suitable surface charge may protect nucleic acid from nuclease degradation and promote gene delivery efficiency, thus increasing exogenous gene expression level in skeletal myocytes for applicable purpose. However, it is still difficult to find and design such appropriate non-viral materials to compress pDNAs for intramuscular gene delivery. In this proof-of-concept study, based on the performances of PEI/pDNA (N/P = 0.5) and EGCG/pDNA (molar mass ratio = 10), we first reveal that the critical principle for material/pDNA complex benefitting intramuscular gene delivery/expression is to keep the complex negatively charged. Meanwhile, from the results we could conclude that negatively charged or even neutralized materials with strong affinity to nucleic acids and/or other properties suitable for gene delivery are better choices and more promising than cationic materials in muscle-based gene delivery systems. The principle and strategy explored in this study may be advantageous to screen and design materials to develop powerful intramuscular gene expression systems for clinical applications. The novel L/E/G approach established here has already showed the applicable potentials, evident from the accelerated body growth and the enhanced GHRH concentration of mice.
